# Assessment of a Syndromic Surveillance System Based on Morbidity Data: Results from the Oscour® Network during a Heat Wave

**DOI:** 10.1371/journal.pone.0011984

**Published:** 2010-08-09

**Authors:** Loïc Josseran, Anne Fouillet, Nadège Caillère, Dominique Brun-Ney, Danièle Ilef, Gilles Brucker, Helena Medeiros, Pascal Astagneau

**Affiliations:** 1 Department of Alert Coordination and Regions, French Institute for Public Health Surveillance, Saint Maurice, France; 2 Assistance Publique des Hôpitaux de Paris, Paris, France; 3 Groupement d'Intérêt Public (GIP) Esther, Paris, France; 4 Department of Public Health, Pierre and Marie Curie University School of Medicine, Paris, France; University of Cape Town, South Africa

## Abstract

**Background:**

Syndromic surveillance systems have been developed in recent years and are now increasingly used by stakeholders to quickly answer questions and make important decisions. It is therefore essential to evaluate the quality and utility of such systems. This study was designed to assess a syndromic surveillance system based on emergency departments' (ED) morbidity rates related to the health effects of heat waves. This study uses data collected during the 2006 heat wave in France.

**Methods:**

Data recorded from 15 EDs in the Ile-de-France (Paris and surrounding area) from June to August, 2006, were transmitted daily via the Internet to the French Institute for Public Health Surveillance. Items collected included diagnosis (ICD10), outcome, and age. Several aspects of the system have been evaluated (data quality, cost, flexibility, stability, and performance). Periods of heat wave are considered the most suitable time to evaluate the system.

**Results:**

Data quality did not vary significantly during the period. Age, gender and outcome were completed in a comprehensive manner. Diagnoses were missing or uninformative for 37.5% of patients. Stability was recorded as being 99.49% for the period overall. The average cost per day over the study period was estimated to be €287. Diagnoses of hyperthermia, malaise, dehydration, hyponatremia were correlated with increased temperatures. Malaise was most sensitive in younger and elderly adults but also the less specific. However, overall syndrome groups were more sensitive with comparable specificity than individual diagnoses.

**Conclusion:**

This system satisfactorily detected the health impact of hot days (observed values were higher than expected on more than 90% of days on which a heat alert was issued). Our findings should reassure stakeholders about the reliability of health impact assessments during or following such an event. These evaluations are essential to establish the validity of the results of syndromic surveillance systems.

## Introduction

From the time John Graunt published the first epidemiological analysis in 1662 until recently, data recording was limited to paper-based modalities [1]. The Réseau Sentinelles® in 1984, set up in France using the Minitel® (French electronic network), first demonstrated the utility of electronic data recording for routine infectious disease system alerts and feedback transmission for general practitioners [2]. With improvements in electronic technologies, the concept of syndromic surveillance, based on non-specific disease data recorded routinely by healthcare professionals [3]–[5] and transmitted automatically via the Internet [6], has emerged as a valuable resource.

Several syndromic surveillance systems have been developed and deployed worldwide in response to bioterrorist threats [4], [7], [8]. However, syndromic surveillance systems geared towards a public health approach (not limited to bioterrorism) are of growing interest [7], [9], [10]. This method has potential for a number of applications, such as monitoring environmental health effects (heat waves, cold spells, and carbon monoxide poisoning) and infectious diseases (influenza, gastroenteritis, and viral meningitis) [6], [10]–[12]. Evaluation of new public health tools is infrequently reported, however, and should be accorded greater importance [3], [13]–[16]. Although gold standards are lacking, it is important to assess the quality of data, which are increasingly used by decision makers to evaluate public health threats [17].

In July 2004, the French National Institute for Public Health Surveillance (Institut de Veille Sanitaire - InVS) set up a syndromic surveillance system based on three data sources: emergency departments (ED), emergency General Practitioners Service (SOS Médecins) and mortality data from city registry offices. Data are collected daily and automatically from the three sources [5]. The surveillance system based on ED, called Oscour®, has been deployed across the country (including in French overseas territories) but has yet to be fully evaluated. This paper aims to evaluate this system in the context of the heat wave that occurred in France in the summer of 2006.

## Methods

### Ethical approval

The use of this database for epidemiological studies has been authorized by the French National Commission for Data protection and Liberties (CNIL) and has received an agreement number, 1015929, in accordance with Act N°78–17 of 6 January, 1978 on Data Processing, Data Files and Individual Liberties.

### Data collection

#### ED data

Following the 2003 heat wave in France, a volunteer surveillance network of hospital emergency departments was set up to collect individual patient data on a daily basis. Details of this network have been published elsewhere [5]. For this study, 15 EDs (out of 32) were selected according to two criteria: location in Ile-de-France (Paris and the surrounding area), and a data set available for 2005 and 2007. This corresponded to 38.7% of all ED visits logged in the Ile-de-France and represented a population of 6.3 million individuals (10% of the entire French population). For each patient, the following data were collected: age, gender, zip-code, reason for emergency admission, main medical diagnosis based on the tenth edition of the International Classification of Diseases (ICD-10), and whether the patient was admitted for hospitalization after discharge. Encrypted data from the previous midnight-to-midnight 24-hour period were transmitted daily to InVS over the Internet using FTP (File Transfer Protocol). The process is automatic and includes: conversion of data from XML format to SAS format, correction of integrated data where necessary (zip code) and calculation of complementary variables (age from date of birth). All hospital discharge records were anonymous and were processed in line with national patient confidentiality rules. The current study was conducted from June 1 to August 31, 2006, based on criteria defined in the National Heat Wave Plan [18].

#### Meteorological data

Daily temperatures (minima and maxima) were obtained from Météo France (National Weather Forecast) and collected by a network of 4 meteorological stations located in the Ile-de-France between 1^st^ June and 31^st^ August, 2006.

#### Heat wave and alert period: the gold standard

Following the 2003 heat wave, a biometeorological alert indicator was defined. It was based on the maximum and minimum temperatures recorded for the target area (Paris and the surrounding region). These were constructed from a study of the relationship between mortality and temperature over three consecutive days over a 30-year period. Two alert thresholds were defined for Ile-de-France as 31°C (T max) and 21°C (T min), corresponding to an increased risk of mortality of 50%. The complete methodology has been published elsewhere [19]. The gold standard for the current study corresponds to this biometeorological alert indicator. The heat wave period was defined by considering the days on which both alert thresholds (T° max and T° min) were breached in Ile-de-France. In total, 13 days were classified as “On Alert Periods” (ONAP), (1^st^ to 4^th^ and 17^th^ to 25^th^ July 2006). The “Off Alert Periods” (OFAP) (alert thresholds not breached) included June 1^st^ to 30^th^, July 5^th^ to 16^th^ and July 26^th^ to August 31^st^.

### Data evaluation

#### Data quality

The percentage of “unknown” or “mis-coded” responses was analyzed for the overall period. Data were compared between the ONAP and OFAP periods using a T-test.

#### Stability

The evaluation of data transmission stability (amount of time that the system was fully operational) was based on comparisons between the expected number of data sets sent daily by hospitals and the number of data sets actually received daily from hospitals by InVS. The expected data set transmitted was based on the number of participating EDs, calculated on the basis of the length of the surveillance period in days (number of daily ED data sets ×92 days of surveillance).

#### Flexibility

The flexibility of a surveillance system is defined as the ability of the system to change as needs change, including the adaptability of the system to shift from outbreak detection to outbreak management. The study period extended from July 2004 until April 2009, in order to observe different uses of such surveillance. All of these uses were categorized according to the main syndrome and the type of situation being monitored (routine or exceptional).

#### Timeliness

The timeliness was calculated as the number of hours between the daily data set closing time and the publication of the bulletin for public health authorities. The considered day was defined as the D-Day. The different steps were taking into account (data recording, data transmission, data processing, data analysis, bulletin publication)

#### Cost

The cost for InVS was estimated based on daily use profiles. This estimation was based on annual salaries, including benefits, for personnel involved in the surveillance program in the summer of 2006 and was computed on the basis of time taken to build and operate the system. The costs of IT equipment and both software development and modifications needed to deploy the system in 2004 were also taken into account. These costs were divided by the number of days between 1^st^ July 2004 and 31^st^ August 2006.

### Data analysis

#### Days with an elevated number of cases of heat-related syndromes

Thresholds for the daily numbers of each syndrome were based on a calculation using two historical data sets (summer 2005 and 2007, for both no heat wave was recorded) and on ED data. The computation was based on an algorithm adapted from Semenza [20]. Expected numbers of each syndrome or group of syndromes per day and per age group were calculated over the three summer months (June, July, August) based on the 2005 and 2007 data sets. A 95% confidence interval was calculated for each case. The observed daily counts were considered significantly different from the expected values if they fell outside the 95% confidence limits [21].

#### Days with an elevated number of visits per day

Sensitivity, specificity and positive predictive value were calculated. A true positive was defined as the number of above-threshold days in terms of the number of visits during ONAP. Performances were calculated for three age groups: 15–74 yrs (young adults), 75 yrs and older (elderly), and all adults.

#### Sensitivity, specificity, positive predictive value and correlation coefficient for heat related syndromes and ED visits

**Figure 1:**
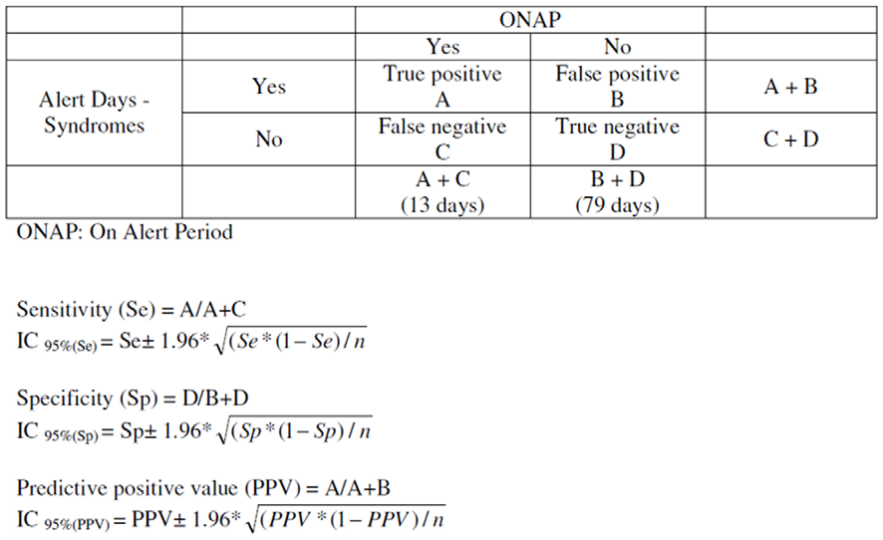
Formulas for computing parameters for Oscour® Syndromic surveillance system.

In a previous work, we identified the most relevant syndromes for evaluating the health burden of heat waves through syndromic surveillance in real time [9]. Four different diseases were selected: hyponatremia, dehydration, malaise, and hyperthermia. Sensitivity refers to the proportion of days that exhibited elevated heat-related disease counts detected by the surveillance system during ONAP (reported cases correctly classified) [22]. Specificity refers to the proportion of days with normal numbers of heat-related diseases during OFAP, and positive predictive value (PPV) refers to the number of days with a significant count of heat-related visits during the ONAP among the total number of days with a significant count of heat-related visits. A true positive (for a given syndrome) was defined as the number of days above threshold in terms of both number of syndromes and temperature (Figure 1). Based on these metrics, syndrome groups were defined and calculated for all three age groups.

A Pearson correlation coefficient was calculated between the daily maximum temperature and the number of syndrome and syndrome combinations observed on the same day.

## Results

During the surveillance period (June 1, 2006, to August 31, 2006), 179,555 patient visits were recorded from participating EDs. The average number of visits was 1,952/day (mean per emergency department  = 131; range 38–260). A total of 26,436 visits were recorded (daily mean  = 2,033.4 visits, mean per ED  = 136 visits, range  = 56–228 visits) for the ONAP, and 153,121 for the OFAP (daily mean  = 1,938.1 visits, mean per ED  = 130 visits, range  = 38–260 visits). During ONAP, the number of visits per day increased significantly among the elderly (172.3/day vs. 157.9/day, *p*<0.05 - Table 1). The average numbers of syndromes per day used for the evaluation of performance are presented by age group and by alert period in Table 1. With respect to heat related diseases, a significant increase in the number of cases during ONAP by age group was observed. Hyperthermia was significantly more frequent among younger adults, while hyponatremia and dehydration were more frequent among the elderly, and malaises were more frequent for all age groups (Table 1).

**Table 1:** Average number of syndromes per day according to age groups and alert period.

	15-74 yrs old	75 and +	All
**ED Visits**ONAPOFAP	1,423.51,344.5	172.3 *157.9	1,595.81,502.3
**Malaises**ONAPOFAP	35.2 ***22.7	11.4 ***7.5	46.6 ***30.2
**Hyperthermia**ONAPOFAP	0.9 *0.4	0.30.1	1.2 *0.5
**Hyponatremia**ONAPOFAP	0.80.4	2.7 **0.7	3.5 **1.1
**Dehydration**ONAPOFAP	0.40.2	2.7 **0.6	3.1 **0.8

Oscour® Network - Paris Area, June 1^st^ to August 31^st^ 2006.

*: *p*<0.05,

**: *p*<0.01,

***: *p*<0.001.

ONAP: On Alert Period; OFAP: Off Alert Period.

### Data quality

Data quality evolved slightly and did not vary significantly throughout the period. Age, gender and outcome were recorded in almost all cases. The proportion of visits with missing or miscoded diagnosis was 37.5% and this was largely due to 4 EDs, where 90% to 100% of records had missing or miscoded diagnoses. Five EDs had more than 93% of records fully complete. Severity was missing or miscoded in 14.21% of records for the entire period and a significant decrease in miscoding was observed during ONAP (13.41% vs. 14.35%, *p*<0.05).

### Stability

The expected number of data sets transmitted was: 15 daily ED data set ×92 days in the surveillance period  = 1,380 expected data sets. In the end, 1,373 data sets were recorded in time, which corresponded to a stability of 99.49%. Seven hospitals were affected by a mis-transmission that occurred on seven different days falling outside the ONAP. During ONAP, the stability was 100% (195 expected data sets and 195 received), and the stability for the OFAP was 99.40% (1,185 data sets expected, 1,178 received).

### Flexibility

Table 2 shows the different syndromes or situations in which the system was used in either routine or emergency mode. Fields of infectious disease and environmental health are covered, as well as industrial accident health impact assessments and stakeholder feedback. Following the situations, results were published weekly (influenza, bronchiolitis), daily (heat wave, cold spell) for a limited period (hurricane health impact assessment) or for the entire year (carbon monoxide poisoning).

**Table 2:** Syndromes or situations monitored using Oscour ® Network. July 2004 to April 2009– France.

Syndromes or situations	Monitored period
**Infectious diseases**	
Influenza	Winter
Bronchiolitis	Fall and Winter
Viral meningititis	All year
Gastro-enterititis	Fall and Winter
Measles	All year
Dengue	Winter ED located in French over seas departments
**Environmental health**	
Asthma	Spring, Summer, Fall
Cold weather impact	Winter
Hot weather impact	Summer
Carbon monoxide poisoning	All year
Extreme weather event (hurricane, floods, heat)	All year
**Others**	
Industrial accident impact	All year
Stakeholders reassurance	All year
Mass gathering (healthMonitoring)	All year

ED: Emergency Department.

### Timeliness

**Figure 2:**
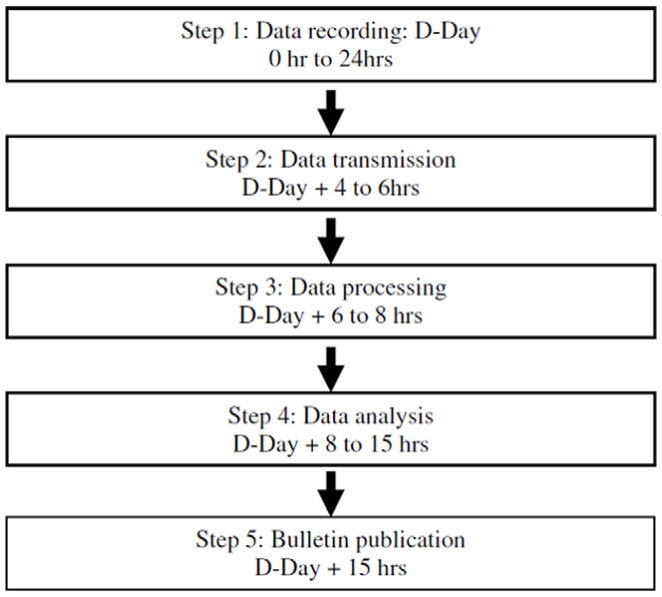
Timeline: Oscour ® Network - Paris area, Summer 2006.

Data for a single day were recorded from midnight to midnight. Figure 2 illustrates the different steps from data transmission to bulletin publication. The transmission step included: time needed for data transmission from hospital to InVS through regional level which gathered all local ED data sets, the time taken by data processing (transcription data from XML format to SAS format, correction of integrated date if necessary or calculation of complementary variables, like age from date of birth). The longest step concerned data analysis which included time for data validation and the report writing by epidemiologist. A maximum of 15 hours is taken for the all process and it is typically completed by 3 pm every day during the ONAP to provide dashboards to health authorities.

### Cost

Three employees maintained the system daily: a senior epidemiologist (annual cost €70,000 and 50% of their time), a junior statistician (annual cost €35,000 and 100% of their time) and a senior data processing expert (annual cost: €70,000 and 10% of their time). The human daily cost was estimated at €211 ((35,000×2) +7,000)÷365). In July 2004, the initial IT equipment and software development investment needed to set up the system totaled €60,000. For the study period, the average cost per day was estimated at €287 (€211 human + €76 IT (€60,000÷790 days (July 1^st^, 2004 to August 31^st^, 2006)).

### Sensitivity, specificity positive predictive value and correlation coefficient

Concerning the indicator “ED Visits,” the best sensitivity was for the elderly, with a value of 0.38 (Table 3). Considering each syndrome separately, sensitivity was good among the elderly for malaise (0.85), dehydration (0.77) and hyponatremia (0.77) (Table 3). Among young adults, the highest sensitivity was 0.69 for malaise (Table 3). Considering syndromes by group, sensitivity was high for groups of at least three syndromes. The best sensitivity (0.92) was obtained with for different groups (all syndromes, Malaise-Hyperthermia-Dehydration, Malaise-Hyperthermia-Hyponatremia, Malaise-Hyponatremia-Dehydration) and concerned different age groups (“All adults”, “75 and above”) (Table S1).

**Table 3:** Sensitivity, specificity, positive predictive value and correlation coefficient of syndromes and ED visits according to age group, compared with ONAP.

All adults	A/D	Sensitivity (CI 95%)	Specificity (CI 95%)	PPV (CI 95%)	Corr Coeff
**ED Visits**N = 139,433	1/77	0.08 (0.02–0.13)	0.97 (0.94–1.00)	0.33 (0.23–0.43)	0.44
**Dehydration**N = 133	10/63	0.77 (0.68–0.86)	0.80 (0.72–0.88)	0.38 (0.28–0.48)	0.47
**Hyperthermia**N = 53	5/72	0.38 (0.28–0.48)	0.91 (0.85–0.97)	0.42 (0.32–0.52)	0.52
**Malaise**N = 3,711	11/58	0.85 (0.78–0.92)	0.73 (0.64–0.82)	0.34 (0.25–0.43)	0.58
**Hyponatremia**N = 157	9/68	0.69 (0.60–0.78)	0.86 (0.79–0.93)	0.45 (0.35–0.55)	0.53
**15–74 yrs**					
**ED Visits**N = 124,717	2/73	0.15 (0.08–0.22)	0.92 (0.87–0.97)	0.25 (0.16–0.34)	0.43
**Dehydration**N = 31	0/76	0.00 (0.00–0.00)	0.96 (0.92–1.00)	0.00 (0.00–0.00)	0.25
**Hyperthermia**N = 44	4/72	0.31 (0.21–0.41)	0.91 (0.85–0.97)	0.36 (0.26–0.46)	0.51
**Malaise**N = 2,872	9/58	0.69 (0.60–0.79)	0.73 (0.64–0.82)	0.30 (0.21–0.39)	0.57
**Hyponatremia**N = 52	3/73	0.23 (0.14–0.32)	0.92 (0.87–0.98)	0.33 (0.24–0.42)	0.32
**75 and above**					
**ED Visits**N = 14,716	5/64	0.38 (0.28–0.48)	0.81 (0.73–0.89)	0.25 (0.16–0.34)	0.22
**Dehydration**N = 102	10/67	0.77 (0.68–0.86)	0.85 (0.78– 0.92)	0.45 (0.35–0.55)	0.46
**Hyperthermia**N = 9	2/74	0.15 (0.07–0.23)	0.94 (0.89–0.99)	0.29 (0.19–0.39)	0.30
**Malaise**N = 839	11/59	0.85 (0.78–0.92)	0.75 (0.66–0.84)	0.35 (0.26–0.44)	0.31
**Hyponatremia**N = 105	10/63	0.77 (0.68–0.86)	0.80 (0.72–0.88)	0.38 (0.28–0.48)	0.49

Oscour® Network, Paris area, June 1^st^ to August 31^st^ 2006

PPV: Predictive Positive Value – CI: Confidence Interval – ONAP: On Alert Periods

ED: Emergency Department

Corr. coef.: correlation coefficient between the daily number of visits in ED and the maximum temperature recorded the same day.

A: true positive day (number of days with a significant count of heat-related visits during the ONAP)

D: true negative day (number of days with a non-significant count of heat-related visits during the OFAP)

Specificity for the “ED Visits” indicator was 0.97 for the group “All adults”. Among syndromes taken individually, specificity was good for “Dehydration” among young adults (0.96) and this corresponded to the best value. Hyperthermia appeared as a very specific indicator for the different age groups. The best specificity among groups of three syndromes was found for the group “Hyperthermia-Hyponatremia-Dehydration” (Table S1). Inversely, Malaise and syndrome combinations including Malaise had the lowest specificity (Tables 3 & S1).

The best PPV with syndromes taken alone (0.45) was obtained for “Hyponatremia” for the group “All adults” and “Dehydration” for elderly. Among younger adults, the best PPV (0.36) was obtained for “Hyperthermia”. Two age groups (elderly and “All adults”) reached 0.56 (the best PPV): for “Hyponatremia-Dehydration” (Table S1).

Results of the last three combinations of 2 syndromes (those including “malaise”) are not presented here. They presented similar performances to the combinations of 3 syndromes including “malaise”.

The best CC with syndromes taken alone was for “Malaise” and the two age groups “All adults” (0.58), “15–74 years” (0.57). For the age group “75 and above” the best CC was 0.46 for “Dehydration” (Table 3). For syndromes combination, CC was between 0.38 and 0.67 according to age groups and syndrome groups (Table S1).

## Discussion

This study confirms the ability of a syndromic system based on hospital emergency activity to detect the health impact of a heat wave, since more than 90% of hot days on National Heat Wave Plan alert exhibited observed values for syndrome incidence that were higher than expected. The study was based on standard criteria for evaluating quality and performance including stability (system response rate), timeliness, flexibility, and effectiveness (sensitivity/specificity and positive predictive value). We observed a data transmission response rate (stability) of nearly 100%. This result indicates that the Oscour® Network successfully acquires almost all the data needed for analyses on a daily basis. However, the amount of data transmitted daily and the automation of data recording makes it impossible to use any data transmission method besides the Internet. If a hospital were to lose its Internet connection, the system would fail; during a crisis, this situation is not acceptable for public health surveillance purposes. Therefore, it is necessary to devise alternative solutions for data transmission that can be implemented as a backup in case of network failure.

The Oscour® Network calculates a daily analysis by 3:00 pm on the day after data acquisition (Figure 2). This should allow sufficient time for stakeholders to manage, in near real time, the response to early warnings and to deal with any public health situation in a timely fashion. In comparison, the flux of data generated by other surveillance systems is scheduled by day or by week [23], [24]. It is most likely difficult get below 15 hrs of response delay due to the Oscour® technical architecture. A complete automation of the entire process (except analysis) may save 2 or 3 hours. Data recording and analysis in perfect real time, as proposed in some specialized systems, is probably not necessary for public health surveillance [25]. Direct communication with emergency physicians (phone, e-mail) based on qualitative analyses from physicians seems to be more valuable than information collected and analyzed continuously. Short timelines are key for the acceptance of the system by ED staff. We consider the network architecture to be a crucial part of syndromic surveillance. Though it is a difficult parameter to evaluate, it is necessary to include this criterion in any evaluation of syndromic surveillance systems. Moreover, it could be considered as a proxy for evaluating the true acceptance of the system by ED staff. Syndromic surveillance is frequently described as being well-accepted by participants, due to the fact that data transmission is automatic and no extra work is required by staff [6]. In addition, the daily cost of such a system is low, €287/day. In comparison, the annual cost of the syndromic surveillance system (NHS Direct) operated in England and Wales was estimated at around $280,000 ($767/day) [3]. The cost of the surveillance system developed by the French Army was estimated at €235,000/year [26].

**Figure 3:**
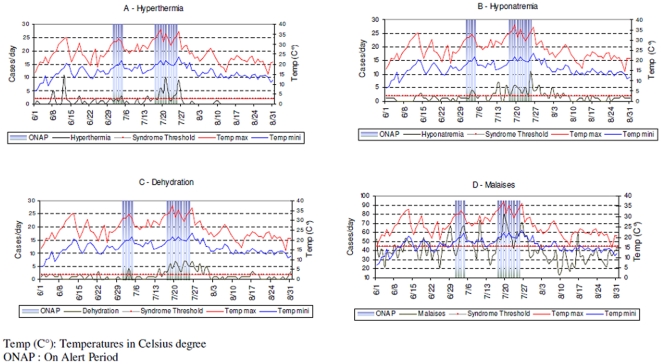
Daily follow-up of temperature (max and min) and syndromes – Oscour® Network Paris area – June 1^st^ to August 31^st^ 2006.

Performance of the system, based on the total number of visits per day, may be limited in the context of heat waves [27]. This point reinforces the need for monitoring pooled diseases and stratifying by age groups. Our data support the idea that monitoring groups of syndromes, instead of separate disease or symptoms, improves the effectiveness of both surveillance and alert management. Concerning the elderly and the total adult populations, our data showed that a three-syndrome combination (including malaises, hyperthermia, and dehydration) would be the most effective grouping strategy. For young adults, a two syndrome combination (including hyperthermia and dehydration) would be the most appropriate. Hyponatremia appears to be a sensitive indicator of heat-related health effects, but this should be understood as a harmful effect of prevention (Figure 3). Therefore, temporal fluctuations of this indicator should be followed only during a subsequent heat event in order to assess the effects of prevention measures. In the case of an increase of hyponatremia during hot periods, recommendations for appropriate prevention should be disseminated promptly.

Malaise is a particular indicator, it is sensitive but its specificity and PPV are low and our experience shows it increased or decreased quickly during various event (hot weather, mass gathering…). Figure 3 supported that point. The daily number of “malaise” increased sharply during ONAP but also the 10^th^ of July (Gay Pride in Paris, 800,000 participants). Therefore it seems difficult to use this indicator alone or in a combination of 2 syndromes.

Our results illustrate that among days with a significant heat related visits, nearly 60% of them were situated during OFAP. Most occurred during the first two weeks of June (hyperthermia), at the end of July (dehydration), between the two ONAPs, and at the end of July (hyponatremia and malaises). These peaks should not be considered false positives, but are partly due to the impact of hot weather, even if the ONAP threshold was not exceeded (but it was close). They also represent the delayed impact of hot weather (with peaks at the end of July). This aspect raises the issue of whether the gold standard used for this evaluation is appropriate. The alert threshold was based on mortality according to temperature, whereas the surveillance system recorded morbidity data. This could explain why disease peaks were detected before and after ONAP, since air temperature levels were sufficiently elevated for people to become ill but not high enough to cause death.

Results of the surveillance are used daily throughout the summer period as complement of the National Heat Wave Plan which is based on observed and forecast temperatures. This plan helps to define ONAP and OFAP but it is not tailored to estimate the burden of heat on population. Moreover, during ONAP, prevention measures are applied and modified the heat effect on population health. Then the expected, and not linear, relations between temperatures and health effect may be changed and made more useful specific public health surveillance using morbidity.

This study presents several limitations. The method adapted from Semenza to define threshold alerts for syndromes does not take into consideration several parameters. Modeling is needed in order to consider complementary elements, limited healthcare availability (GP's) during France's summer vacation period that could have an effect on ED activity, day effect (ONAP, OFAP). Our evaluation is focused on a limited time period that included only one heat wave, which probably led to an overvaluation of stability and other parameters. More research is needed to determine the criteria that should be evaluated in other situations. Confirmation of these results by other studies conducted under the same weather conditions may also become necessary. Furthermore, the representativeness of the network during the study should be considered a serious limitation. We analyzed less than 40% of all ED regional activity. However, the objective of such surveillance systems is to identify the health effects of events as soon as possible, not to assess the impact of an event [28]; this should be explored by other specific studies. For the Ile-de-France, the limited number of regional hospitals does not appear to present a difficulty for evaluating performance. As this region is relatively small, weather conditions were homogenous and the health consumption behavior of the population was uniform. Therefore, with EDs distributed across the region, the system was able to correctly detect the health effects of heat waves. We observed a loss of 35% of all final diagnoses due to physician non-compliance, and this could have contributed to a reduction in the power of our analysis. The flexibility of the system (Table 2) can help improve data quality through frequent interactions and the direct involvement of ED staff in public health surveillance.

Evaluation is an important component in the development of a health surveillance system, but in practice, for various reasons, this step is often omitted or unsatisfactory [14], [15]. Syndromic surveillance could be considered a new method of health surveillance, and evaluations of such systems seem particularly important. Although evaluations are rare, they are the only way to demonstrate the real utility of syndromic surveillance. One explanation is the difficulty in fitting classical evaluation framework onto a syndromic surveillance system. Buehler and colleagues first published a framework that was adapted for the evaluation of syndromic surveillance systems, but it is now necessary to define specific criteria to best evaluate such systems [15].

## Supporting Information

Table S1 Sensitivity, specificity, positive predictive value and correlation coefficient of syndrome groups according to age group, compared to ONAP.
(0.07 MB DOC)
