# Supplementary material for: Assessment of a Syndromic Surveillance System Based on Morbidity Data: Results from the Oscour® Network during a Heat Wave
Source: PLoS One. 2010 Aug 9;5(8):e11984. doi: 10.1371/journal.pone.0011984 (PMC2918496; doi:10.1371/journal.pone.0011984)
Supplement: Table S1 — Sensitivity, specificity, positive predictive value and correlation coefficient of syndrome groups according to age group, compared to ONAP. (0.07 MB DOC) [file pone.0011984.s001.doc]

**Table S1.** Sensitivity, specificity, positive predictive value and correlation coefficient of syndrome groups according to age group, compared to ONAP. Oscour ® Network

Paris Area, June 1^st^ to August 31^st^ 2006

| **All adults** | **A / D** | **Sensitivity**  **(CI 95%)** | **Specificity**  **(CI 95%)** | **PPV**  **(CI 95%)** | **Corr. Coef.** |
| --- | --- | --- | --- | --- | --- |
| **All Syndromes**  HyperTh, HypoNa, D, M | 12 / 57 | 0.92 (0.86-0.98) | 0.72 (0.63-0.81) | 0.35 (0.25-0.45) | 0.65 |
| **3 Syndromes**  HypoNa, D, M | 12 / 57 | 0.92 (0.88-0.98) | 0.72 (0.63-0.81) | 0.35 (0.25-0.45) | 0.63 |
| HyperTh, HypoNa, D, | 9 / 69 | 0.69 (0.77-0.92) | 0.87 (0.81-0.94) | 0.47 (0.37-0.57) | 0.65 |
| HyperTh, D, M | 12 / 54 | 0.92 (0.86-0.98) | 0.68 (0.58-0.78) | 0.32 (0.22-0.42) | 0.63 |
| HypoNa, HyperTh, M | 12 / 58 | 0.92 (0.86-0.98) | 0.73 (0.64-0.82) | 0.36 (0.26-0.46) | 0.63 |
| **2 Syndromes**  HyperTh, D | 10 / 65 | 0.77 (0.68-0.86) | 0.82 (0.74-0.90) | 0.42 (0.32-0.52) | 0.58 |
| HypoNa, D | 9 / 72 | 0.69 (0.60-0.78) | 0.91 (0.85-0.97) | 0.56 (0.46-0.66) | 0.57 |
| HyperTh, HypoNa | 8 / 71 | 0.62 (0.52-0.72) | 0.90 (0.84-0.96) | 0.50 (0.40-0.60) | 0.67 |
| **15-74 yrs** |  |  |  |  |  |
| **All Syndromes**  HyperTh, HypoNa, D, M | 9 / 56 | 0.69 (0.60-0.78) | 0.71 (0.62–0.80) | 0.28 (0.19-0.37) | 0.61 |
| **3 Syndromes**  HypoNa, D, M | 9 / 58 | 0.69 (0.60-0.78) | 0.73 (0.64-0.82) | 0.30 (0.21-0.39) | 0.59 |
| HyperTh, HypoNa, D, | 3 / 72 | 0.23 (0.14-0.32) | 0.91 (0.85-0.97) | 0.30 (0.21-0.39) | 0.58 |
| HyperTh, D, M | 9 / 55 | 0.69 (0.60-0.78) | 0.70 (0.61-0.79) | 0.27 (0.18-0.36) | 0.60 |
| HypoNa, HyperTh, M | 9 / 58 | 0.69 (0.60-0.78) | 0.73 (0.64-0.82) | 0.30 (0.21-0.39) | 0.60 |
| **2 Syndromes**  HyperTh, D | 9 / 70 | 0.69 (0.60-0.78) | 0.89 (0.82-0.95) | 0.50 (0.40-0.60) | 0.50 |
| HypoNa, D | 1 / 72 | 0.08 (0.02-0.14) | 0.91 (0.85-0.97) | 0.13 (0.06-0.19) | 0.38 |
| HyperTh, HypoNa | 2 / 76 | 0.15 (0.08-0.23) | 0.96 (0.92-1.00) | 0.40 (0.30-0.50) | 0.60 |
| **75 and above** |  |  |  |  |  |
| **All Syndromes**  HyperTh, HypoNa, D, M | 11 / 62 | 0.85 (0.78-0.92) | 0.78 (0.70–0.86) | 0.39 (0.29–0.49) | 0.50 |
| **3 Syndromes**  HypoNa, D, M | 11 / 62 | 0.85 (0.78-0.92) | 0.78 (0.70–0.86) | 0.39 (0.29–0.49) | 0.49 |
| HyperTh, HypoNa, D, | 10 / 71 | 0.77 (0.68-0.86) | 0.90 (0.84-0.96) | 0.42 (0.32-0.52) | 0.55 |
| HyperTh, D, M | 12 / 62 | 0.92 (0.87-0.98) | 0.78 (0.70-0.86) | 0.41 (0.31–0.51) | 0.43 |
| HypoNa, HyperTh, M | 10 / 63 | 0.77 (0.68-0.86) | 0.80 (0.72-0.88) | 0.38 (0.29–0.47) | 0.44 |
| **2 Syndromes**  HyperTh, D | 10 / 65 | 0.77 (0.68-0.86) | 0.82 (0.74-0.90) | 0.42 (0.32-0.52) | 0.47 |
| HypoNa, D | 10 / 71 | 0.77 (0.68-0.86) | 0.90 (0.84-0.96) | 0.56 (0.46-0.66) | 0.54 |
| HyperTh, HypoNa | 8 / 72 | 0.62 (0.52-0.71) | 0.91 (0.85-0.97) | 0.53 (0.43-0.63) | 0.51 |

PPV: Positive Predictive Value – CI: Confidence Interval – ONAP: On Alert Periods

ED: Emergency Department

HyperTh : Hyperthermia, HypoNa : Hyponatremia, D : Dehydration, M : Malaise

Corr. coef.: correlation coefficient between the daily number of visits in ED and the maximum temperature recorded the same day.

A: true positive day (number of days with a significant count of heat-related visits during the ONAP)

D: true negative day (number of days with a non-significant count of heat-related visits during the OFAP)
